# Beyond traditional models: microfluidic technologies for engineering the human placenta *in vitro*

**DOI:** 10.3389/fbioe.2026.1777568

**Published:** 2026-03-16

**Authors:** Alice Masserdotti, Anna Cargnoni, Rashmi Ramakrishnan, Lisa Muiznieks, Justine Lereculey-Beaumanoir, Ivana Brenta, Michael Gasik, Paola Chiodelli, Antonietta Rosa Silini, Ornella Parolini

**Affiliations:** 1 Department of Life Science and Public Health, Università Cattolica del Sacro Cuore, Rome, Italy; 2 Centro di Ricerca E. Menni, Fondazione Poliambulanza Istituto Ospedaliero, Brescia, Italy; 3 CURAM Research Ireland Centre for Medical Devices, University of Galway, Galway, Ireland; 4 Microfluidics Innovation Center, Paris, France; 5 Seqvera Ltd., Helsinki, Finland; 6 Fondazione IRCCS Casa Sollievo della Sofferenza, San Giovanni Rotondo, Italy

**Keywords:** microfluidics, modeling, placenta, placental models, placenta-on-chip

## Abstract

The human placenta is a highly specialized and dynamic organ that supports fetal development by regulating maternal–fetal exchange, endocrine activity, and immune tolerance throughout pregnancy. Despite its central role in maternal and fetal health, studying human placental physiology and pathology remains challenging due to ethical constraints, limited tissue accessibility, and the complexity of the maternal–fetal interface. Traditional *in vitro* models and animal systems have provided valuable insights but often fail to capture the dynamic, multicellular, and perfused nature of the human placenta. Microfluidic technologies have recently emerged as powerful tools for placental modeling *in vitro*. By integrating controlled fluid flow, three-dimensional architecture, and relevant placental cell types, placenta-on-chip platforms enable a more physiologically relevant reconstruction of the maternal and fetal compartments. These systems support the study of placental barrier function, nutrient and drug transport, endocrine signaling, immune interactions, and responses to pathological stimuli under defined and reproducible and tunable conditions. As the field rapidly expands, a comprehensive synthesis is needed to clarify how these systems complement or surpass existing models and to identify the remaining translational gaps. Reliable microphysiological systems that replicate placental “functionality-on-chip” are essential for global regulatory efforts. The need for clinical data to guide safe and effective use of medicines during pregnancy and breastfeeding has led to recommendations from the International Council for Harmonisation of Technical Requirements for Pharmaceuticals for Human Use to include pregnant and breastfeeding women in clinical trials. This creates an urgent push for early data collection on drug effects in pregnancy and breastfeeding using lab studies. This underscores the need to develop *in vitro* systems that reliably predict the effects of drugs and toxicants on the placenta. This review critically examines current placental models, from conventional two- and three-dimensional cultures and animal models to advanced microfluidic systems. We highlight how microfluidic placental models overcome key limitations of traditional approaches and discuss their applications in developmental biology, pharmacokinetics, toxicology, infection studies, and pregnancy-related disorders. Collectively, emerging evidence suggests that microfluidic placental models are central tools for mechanistic studies and preclinical testing, bridging the gap between reductionist systems and human physiology. Future progress will depend on improving model standardization, incorporating additional cellular complexity and immune components, and aligning microfluidic outputs with clinically relevant endpoints. Advancing these platforms toward greater physiological fidelity and interoperability with multi-organ systems will be critical for translating placental research into improved maternal–fetal health outcomes. Beyond summarizing recent technological advances, this review uniquely positions placenta-on-chip systems within the broader landscape of existing placental models and emerging regulatory needs, highlighting their translational potential for drug safety, developmental toxicology, infection biology, and pregnancy-related disorders.

## 1 Introduction

The human placenta is a highly specialized and complex organ that functions as the critical interface between maternal and fetal circulations, enabling maternal–fetal exchange and pregnancy support. Its unique architecture, consisting of both maternal and fetal components, enables these vital processes to occur without direct mixing of maternal and fetal blood. Throughout gestation, the placenta undergoes continuous morphological and functional adaptations to meet the evolving needs of the developing fetus (Masserdotti et al., 2024).

Despite its pivotal role in fetal development and maternal health, the study of the human placenta remains inherently challenging. Ethical constraints significantly limit *in vivo* investigations, and the anatomical complexity and temporally dynamic nature of pregnancy restrict access to functional placental tissue. Furthermore, the placenta’s developmental complexity complicates research, as findings derived from one gestational stage may not be directly applicable to others (Masserdotti et al., 2024). Conventional *in vitro* and animal models often fall short in capturing the dynamic physiological characteristics of the placenta. Static cell culture systems, although valuable for basic research, lack the three-dimensional architecture and fluid dynamics that are essential for accurately recapitulating placental physiological function (James et al., 2022; Mosavati et al., 2020; Karvas et al., 2022). Similarly, interspecies differences in placental structure and physiology constrain the translational relevance of animal models. Animal models provide valuable insights into human pregnancy and placental biology, with rats and rabbits, usually applied in regulatory reproductive toxicity studies, and mice and rats in mechanistic placental research. However, interspecies differences in placental architecture (for example, humans have hemochorial villous placentas while mice hemochorial labyrinthine placentas), gestation duration (∼280 days in humans and ∼20 days in mice), and endocrine function (humans rely mainly on the corpus luteum for post-implantation hormones, while mice have minimal luteal support) (Carter, 2020; Enders and Blankenship, 1999), limit the translational relevance of these *in vivo* models.

To address these limitations, researchers have increasingly turned to advanced methodologies aimed at more accurately modeling the placental environment. Among these, microfluidic technology has emerged as a particularly promising approach. Microfluidic systems offer highly controllable and tunable platforms that allow investigation of placental biology within a fluidically dynamic microenvironment.

Moreover, microfluidic placental models enable precise regulation of fluid flow, thereby offering the ability to replicate the shear stress conditions experienced by placental cells *in vivo*. These models also facilitate real-time monitoring of cellular responses, nutrient exchange, and transplacental transport of drugs and other substances.

As the field advances, significant efforts are being directed toward enhancing the complexity and physiological fidelity of these models. This review provides a critical examination of placental modeling, contrasting the inherent limitations of conventional approaches with the transformative potential of advanced microfluidic placental systems. We explore their diverse applications in unraveling complex placental physiology and pathology, concluding with an assessment of current hurdles and future directions for this evolving research frontier.

While several recent reviews have described the development of placenta-on-chip systems and microphysiological (MPS) models of the maternal-fetal interface, most have focused primarily on device engineering, individual applications, or specific disease contexts. In contrast, this review provides a cross-scale and translationally oriented perspective that integrates (i) conventional 2D, 3D, and animal placental models, (ii) advanced microfluidic and organoid-on-chip technologies, and (iii) emerging regulatory and clinical needs in maternal–fetal medicine. By positioning placenta-on-chip systems within the broader landscape of existing models and explicitly discussing their potential role in drug safety assessment, developmental toxicology, infection biology, and pregnancy disorders, we aim to better describe the specific added value that microfluidic platforms can offer. We also highlight steps required for standardization, benchmarking, and regulatory qualification, framing placenta-on-chip technologies not only as research tools but as next-generation translational platforms with potential impact on preclinical testing and maternal–fetal health decision-making.

## 2 The human placenta: structure and function

The placenta is a remarkable, transient fetal-maternal organ, fundamental for successful pregnancy by facilitating vital gas and nutrient exchange, waste elimination, and fetal-maternal tolerance (Masserdotti et al., 2024; Benirschke et al., 2012; Yang et al., 2019).

At term, the human placenta typically presents as a flat, round, or oval disc-like organ, measuring approximately 15–20 cm in diameter and 2–3 cm in thickness, with an average weight between 500 and 600 g (Parolini et al., 2008).

**FIGURE 1:**
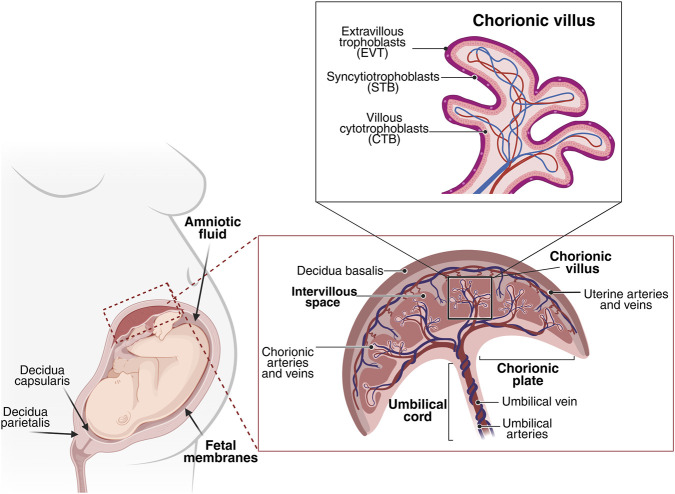
Schematic representation of the human placenta, umbilical cord, and fetal membranes. The umbilical cord inserts into the chorionic plate, the fetal component of the placenta, from which chorionic villi extend into the intervillous space. The maternal component of the placenta is formed by the decidua, which interacts with invading trophoblasts at the site of implantation. Adapted from Masserdotti et al., Frontiers in Cell and Developmental Biology (2024), https://doi.org/10.3389/fcell.2024.1411582.

The placenta is a composite organ (Figure 1), intricately formed from both fetal and maternal components. The fetal contribution originates from the blastocyst and includes the chorionic plate, amniotic and chorionic membranes, and umbilical cord. The functional units for fetal-maternal exchange are called chorionic villi. These are finger-like structures that sprout from the chorionic plate into the intervillous space, a critical cavity where maternal blood circulates in direct contact with the chorionic villi, which contain fetal blood (Parolini et al., 2008; Huppertz, 2008).

The chorionic villi develop progressively from primary to tertiary forms, establish extensive capillary networks, and anchor the placenta to the maternal endometrium.

Microscopically, the villi are covered by specialized trophoblast cells: the outer, continuous, multinucleated syncytiotrophoblasts (STB), which are in direct contact with maternal blood and possess a vast surface area, and the underlying cytotrophoblasts (CTB), which act as stem cells for STB replenishment. The third type, extravillous trophoblasts (EVT), are crucial for invading and remodeling maternal spiral arteries, transforming them into low-resistance vascular channels essential for adequate blood supply (Masserdotti et al., 2024; Griffiths and Campbell, 2015).

The fetal membranes, the amnion (innermost) and chorion, enclose the fetus and its surrounding amniotic fluid, forming a highly specialized protective barrier. The amniotic membrane is a thin, avascular structure composed of an epithelial layer and a mesenchymal layer with an extracellular matrix and sparse stromal cells. It is adjacent to the chorionic membrane, which includes the mesenchymal and trophoblastic regions (Beall et al., 2007; Truong et al., 2023; Parolini et al., 2008).

The umbilical cord, which develops by week 7 of gestation, is the vital conduit connecting the fetus to the placenta. It typically contains two umbilical arteries, that transport deoxygenated blood and waste from the fetus, and one umbilical vein, that carries oxygenated, nutrient-rich blood to the fetus. These vessels are protected by a gelatinous extracellular matrix known as Wharton’s jelly, which prevents vessel compression and torsion (Anzalone et al., 2010; Spurway et al., 2012; Di Naro et al., 2001).

The maternal component is the decidua, formed by the proliferation of endometrial cells post-implantation and divided into three regions: decidua basalis, capsularis, and parietalis. It plays a critical role in anchoring the placenta and establishing an immune-privileged environment to prevent maternal immune rejection of the fetus (Abumaree et al., 2016; Mori et al., 2016).

Physiologically, the placenta acts as a selective barrier, facilitating the transfer of gases (oxygen and carbon dioxide) and nutrients, such as glucose and iron, while removing fetal waste products. The continuous dynamic adaptation of the maternal uteroplacental blood flow and fetal umbilical-placental circulation during pregnancy ensures efficient exchange (Benirschke et al., 2012; Burton and Jauniaux, 2015). It also functions as a major endocrine organ, synthesizing hormones such as human chorionic gonadotropin (hCG) to maintain the corpus luteum, human placental lactogen (hPL) to modulate maternal metabolism for fetal nutrition, and steroid hormones (estrogen, progesterone) essential for pregnancy maintenance. Furthermore, it produces growth factors (i.e., vascular endothelial growth factor, VEGF) critical for angiogenesis, and orchestrates immune tolerance while transferring maternal IgG antibodies to the fetus for passive immunity (Gude et al., 2004; Huppertz, 2020).

Dysregulation of placental development is strongly implicated in severe pregnancy complications, such as preeclampsia and fetal growth restriction, underscoring its pivotal role in maternal and fetal health outcomes (Benirschke et al., 2012).

## 3 Conventional *in vitro* and *in vivo* models of the placenta

Elucidating the mechanisms underlying placenta-associated complications has relied heavily on the development of *in vitro* models that aim to recapitulate placental structure and function. Herein, we will discuss the commonly used models and their characteristics.

### 3.1 Static 2D cell culture

*In vitro* culture of placental cells, including isolated primary cells, genetically modified primary cell lines, or choriocarcinoma-derived cell lines, offers effective tools for studying placental biology, function, and the pathogenesis of pregnancy-related disorders.

Among the different placental cell types, 2D trophoblast cell models are the most widely used to investigate the molecular mechanisms underlying human placental development and trophoblast differentiation.

By isolating primary cytotrophoblasts from human term placentas and culturing them as monolayers on plastic or extracellular matrix to promote their spontaneous fusion, researchers have been able to explore the syncytialization process. This approach has led to the identification of numerous soluble factors, such as epidermal growth factor (Morrish et al., 1987), as well as key regulatory genes and transcriptional regulators (Knöfler et al., 2000; Yu et al., 2002; Rouault et al., 2016), that promote trophoblast syncytialization.

In vitro-cultured primary trophoblast cells have also been applied to investigate the molecular mechanisms of embryo implantation and specifically to identify the regulators of trophoblast invasion. These models contributed to identify the interplay between negative regulator signaling pathways, such as transforming growth factor-β (TGF-β) (Lash et al., 2005), and positive ones, such as activins (Li et al., 2014) and bone morphogenetic protein 2 (Zhao et al., 2018), in orchestrating proper placental formation during implantation. Furthermore, these models have also evidenced that these cells contribute to placental immune defense against pathogens by producing antiviral factors such as type III interferons (Bayer et al., 2016) and immunomodulatory chemokines (Ander et al., 2018), thereby restricting infections like Zika virus and Toxoplasma gondii. Additionally, human trophoblasts also regulate both systemic and local immune responses against *Listeria* monocytogenes infection through modulation of inflammasome signaling pathway (Megli et al., 2021).

Because primary trophoblast cells are challenging to culture, exhibit limited lifespan, and are often contaminated by other cell types (Li and Schust, 2015), researchers often use immortalized trophoblast or choriocarcinoma-derived cell lines as alternatives.

ACH-3P cells, an immortalized line from first-trimester human trophoblasts, are commonly used to study autocrine and paracrine regulation of trophoblast formation (Erlandsson et al., 2020; Nääv et al., 2020). Immortalized HRT-8/Svneo cells, derived from first-trimester chorionic villi explants, serve to investigate EVT invasion and proliferation (Verma et al., 2018). Carcinoma-derived lines such as BeWo, JEG-3, and JAR are widely employed to study placental endocrine and transport functions (Rothbauer et al., 2017). However, placenta cell lines exhibit significant and functionally important differences in DNA methylation profiles (Novakovic et al., 2011) and in transcriptome (Lapehn et al., 2025) compared to primary human trophoblasts, leading to notable functional disparities between the cell lines and their primary counterparts.

Recently, trophoblast stem cells have been derived from first-trimester villous cytotrophoblasts and human blastocysts (Okae et al., 2018), and also from human induced pluripotent stem cells (hi-PSCs) (Wei et al., 2021). These cells give rise to long-term cultures that can differentiate into 3 subpopulations into the three major trophoblast subpopulations (cytotrophoblast, EVT, and STB-like cells) with transcriptomes closely resembling primary trophoblasts. Such systems offer a valuable model to study trophoblast development and related disorders like miscarriage, preeclampsia, and fetal growth restriction.

While placenta cell monocultures remain useful to investigate some aspects of placenta biology, under controlled and simplified conditions, they fail to capture the complex interactions between the various cell types present in placental villi, such as trophoblasts, mesenchymal cells, Hofbauer cells, and endothelial cells. To address this limitation, 2D co-culture models have been developed that mimic a functional placental barrier to study the transcellular transport of nutrients, chemicals, particles, and drugs at the maternal-fetal interface. These multicellular models combine endothelial cells which are part of the fetal vessels, and trophoblast cells (Aengenheister et al., 2018; Fuenzalida et al., 2024). Other models replicate the early placental villus architecture by including villous mesenchymal fibroblasts as part of the villous stromal compartment, in addition to endothelial and trophoblast cells (Kreuder et al., 2020).

Future refinements to significantly improve the physiological relevance of these co-culture models will likely include the incorporation of immune cells—particularly fetal villus macrophages (Hofbauer cells)—as well as flow systems to simulate maternal and/or fetal blood circulation—which have a crucial impact on placental villi formation (Miura et al., 2015).

### 3.2 3D *in vitro* models: placental explants and organoids

Although 2D *in vitro* models provide highly reproducible experimental results, they do not entirely reflect the *in vivo* environment. Placental explants, despite their own inherent limitations, preserve the intact native microarchitecture, as well as cell-cell interactions and paracrine signaling, thus closely mimicking the *in vivo* conditions.

Villous explants from both early and term gestation represent the most commonly used type of placenta explants for studying nutrient transport mechanisms (Sooranna et al., 1999; Baumann et al., 2014), enzyme activity (Boadi et al., 1992), and nutrient and xenobiotic metabolism (Ermini et al., 2021). Explants from first trimester villi, containing both anchoring and floating villi, have been used to study placentation, invasion of EVT, trophoblast proliferation and differentiation. For instance, studies performed on human villous explants of 5–8 weeks’ gestation demonstrated the importance of oxygen tension and oxygen-regulated early events, mediated by transforming growth factor-beta 3 (TGFβ3) via hypoxia inducible factor-1 (HIF-1) transcription factors, to regulate trophoblast differentiation (Caniggia et al., 2000).

Term gestation villous and amnion explants have been used to explore the role of placenta-derived extracellular vesicles (EVs) in maternal-fetal communication (Fitzgerald et al., 2018; Kupper and Huppertz, 2022). Their cargo—including proteins, lipids, DNA, and miRNAs—can influence maternal cell functions and may be altered by exposure to pollutants (Sheller-Miller et al., 2020) or pathological conditions, suggesting their potential as biomarkers of pregnancy disorders. Furthermore, human term chorionic villi explants have been used to evidence the cytotoxic, oxidative and metabolic effects of pollutants such as microplastics on placental cells, highlighting potential risks for maternal and fetal health (de Sousa et al., 2024).

In addition, comparing placental explants from patients with gestational age-matched controls can reveal underlying placental dysfunctions and molecular mechanisms contributing to the development of pathologies such as intrauterine growth restriction (IUGR) and fetal overgrowth pregnancies complicated by diabetes mellitus (DM). For example, reduced levels of placenta matrix metalloproteinases (MMP)-2 and −9, crucial for placental tissue remodeling processes, have been found in IUGR explants compared to controls (Merchant et al., 2004). Decreased placental β-oxidation observed in human placental explants exposed to high glucose conditions suggested that an altered placental lipid metabolism may contribute to increased maternal-fetal lipid transfer and excess fetal growth in some DM pregnancies (Hulme et al., 2019).

Despite numerous attempts to preserve long-term integrity and functionality of placental explants in vitro—such as employing flow culture systems that more closely replicate the dynamic *in vivo* environment compared to static cultures (Kupper et al., 2023)—the optimal timeframe during which the explants maintain their functional and structural integrity remains unclear.

The limitations posed by variability in the shape and size of placental explants—which affect tissue viability and functionality—can potentially be overcome by better standardizing tissue thickness through the use of precision-cut tissue slices (PCTS). These thin, uniform slices, typically generated with a vibratome or microtome and ranging from 100 to 300 μm in thickness, significantly enhance nutrient and gas diffusion efficiency better preserving tissue integrity and metabolic activity (Wang et al., 2025a).

Placental PCTS have been employed in physiology, pathophysiology and drug toxicity studies. For instance, they enabled research on amino acid transport in placental villi and the impact of tissue calcium on amino acid uptake (Karl et al., 1988). They also contributed to findings of insulin and nitric oxide as regulators of glucose transport in human placenta (Acevedo et al., 2005). More recently, data collected from PCTS showed the ability of SARS-CoV-2 to infect and propagate in human placenta (Fahmi et al., 2021). Additionally, these slices revealed the inhibitory effects of toxicants like nicotine and acetaldehyde, as well as drugs like salicylate and flufenamic acid, on amino acid and sulfate transport, respectively—potentially disrupting fetal growth and electrolyte balance (Fisher et al., 1984; Shennan and Russell, 1991). Studies on slices from alcohol-dependent women at different pregnancy stages showed reduced placental protein synthesis (Tal et al., 1985). Despite their promise as 3D placental models, key challenges persist in standardizing slicing methods, culture medium composition, and reliable viability assessments. Standardized protocols could enhance data consistency, comparability, and the feasibility of long-term studies—currently limited to a maximum of 2 weeks in one study (Yamada et al., 2016).

Placenta organoids constitute another three-dimensional *in vitro* model that enable the investigation of placental function and the mechanisms underlying gestational diseases. They have been successfully generated from cytotrophoblasts from placental tissues collected across all three trimesters of pregnancy (Yang et al., 2022; Turco et al., 2018; Haider et al., 2018).

Notably, naive trophoblast stem cells have also been used to develop these models (Karvas et al., 2022), establishing trophoblast-like organoids capable of differentiating into STB and EVT, thereby faithfully recapitulating the developmental processes of placental villi observed *in vivo*. These organoids demonstrate proliferative and self-renewing properties, and can remain viable for over 5 months (Li et al., 2023). However, trophoblast-like organoids cannot completely recapitulate the complexity of the placenta villi which contain also additional components such as stromal cells, pericytes, fibroblasts and vascular cells.

Building upon these advances, placenta-like organoids including both the trophoblast and vascular lineages have been obtained from human-induced pluripotent stem cells (hiPSCs). These more complex organoids reproduce placental villous-like structure, resembling the key features of first-trimester human placenta in terms of cellular components, and secretory function (Cui et al., 2022).

The development of trophoblast-like organoids presents new possibilities for *in vitro* drug testing to assess their potential teratogenic effects and may provide valuable insights into the underlying mechanisms of gestational diseases, such as preeclampsia. To further enhance their physiological relevance and applicability, recent studies have also focused on addressing structural limitations of these models. A common challenge with current trophoblast organoid models is their inverted polarity, where STB develop on the interior rather than the outer surface of organoids, contrary to their physiological localization on the outer layer of placental villi. This reversed organization can reduce the accuracy of these models in replicating placental functions, especially those involving pathogen defense and nutrient transfer. Encouragingly, recent research has started to overcome this limitation by culturing trophoblast organoids in suspension, which stimulate STB to form on the exterior, better mimicking their natural polarity (Yang et al., 2024).

### 3.3 Animal models of placenta

Animal models offer valuable insights that enhance our understanding of human pregnancy and placental biology, however, caution is warranted when extrapolating these findings, as no animal model fully replicates the structure and function of the human placenta.

A major limitation is the interspecies significant variability in placental structure. In particular, the placental interface between maternal and fetal blood differs widely across species. Based on histology, humans and rodents have a highly invasive hemochorial placenta, with direct contact between maternal blood and chorion. While primates have villous placentas with a single trophoblast layer, rodents exhibit labyrinthine placentas with multiple trophoblast layers (Furukawa et al., 2014). Furthermore, many differences exist also in relation to gestation length and number and size of fetuses. Consequently, the translatability of placental studies from laboratory animals to humans is challenging, and there is lack of “an ideal animal model” that captures all aspects of human pregnancy and fetal development.

Among the available animal models, mice and rats are the most used species due to their relatively low costs, ease of maintenance, the possibility to generate many genetic manipulated models along with inbreed strains, and a long tradition in scientific research. However, in many non-human mammals, maternal and fetal blood flows are arranged in a countercurrent manner, which maximizes concentration gradients for placental exchange. In contrast, the human placenta operates as a concurrent-like exchange system, in which maternal blood pools within the intervillous space, allowing partial equilibration with fetal blood and thereby limiting concentration gradients (Wilkening and Meschia, 1992; McNanley and Wood, 2008).

Given the wide range of animal models developed to replicate both fetal diseases and gestational disorders—such as intrauterine growth restriction (IUGR), preeclampsia, gestational diabetes, and recurrent miscarriage—this section focuses on models of preeclampsia. This condition was selected not only because it is among the most extensively investigated gestational disorders, but also because its severity and complex pathophysiology make it a representative example to illustrate how animal models can provide valuable insights into human pregnancy and placental biology, while also highlighting their inherent limitations. Because spontaneous preeclampsia rarely occurs in animals, a variety of experimental models have been established through surgical, pharmacological, immunological, or genetic manipulations applied before or during pregnancy, with the aim of recapitulating key features of the human disease.

One widely used model of preeclampsia is the reduced uteroplacental perfusion (RUPP) model, which is performed by clipping the lower abdominal aorta above the iliac bifurcation and the main uterine branches on the left and right ovarian arteries during midgestation of Sprague-Dawley rats (Crews et al., 2000). The RUPP model displays many common characteristics of the second phase of human preeclampsia, including hypertension, proteinuria, increased plasma protein levels, and altered placental angiogenic factors (Morton et al., 2019). This model has been useful in revealing key features of preeclampsia, such as the role of reactive oxygen species and mitochondrial dysfunction in mediating hypertension (Vaka et al., 2018), as well as demonstrating the efficacy of chronic treatment with VEGF in increasing endothelium-dependent relaxation in carotid arteries and resolve the hypertension (Gilbert et al., 2010). However, the RUPP model also reduces blood flow to other organs, such as the heart, stomach, kidneys, and intestines, potentially affecting cardiac output and hemodynamic. Additionally, since placental blood flow is permanently blocked, this model cannot evaluate treatments aimed at improving uteroplacental perfusion.

Another approach to mimic preeclampsia is by reducing nitric oxide (NO) production through the inhibition of NO synthase in pregnant rats (Molnár et al., 1994). When applied at various stages of pregnancy, this model leads to symptoms similar to those of preeclampsia, including high blood pressure, proteinuria, thrombocytopenia, and IUGR. However, the clinical relevance of this model is limited, as studies on the role of NO in women with preeclampsia have produced inconsistent findings. Some reports describe reduced NO bioavailability (López-Jaramillo et al., 2008; Socha et al., 2022). or impaired endothelial NO synthase (eNOS) activity associated with endothelial dysfunction, while others show unchanged or even elevated levels of circulating NO metabolites (Acauan Filho et al., 2016; Adu-Bonsaffoh et al., 2015), possibly reflecting compensatory responses or differences in disease stage, sample type (plasma vs. placental tissue), and analytical methods. This variability makes it difficult to conclude that systemic NO deficiency is a universal or primary driver of human preeclampsia, thereby limiting the direct translational value of NO synthase inhibition models.

Transgenic animal models have emerged as promising tools for studying preeclampsia, as they display a wide range of clinical manifestations observed in humans. Notably, transgenic mouse (Takimoto et al., 1996) and rat (Bohlender et al., 2000) models, created by crossbreeding animals expressing human renin and human angiotensinogen, develop several preeclampsia-like features. These include hypertension, albuminuria, end-organ damage, reduced litter sizes, elevated levels of circulating autoantibodies against the angiotensin II type 1 receptor (AT1-AAs), and increased vasoconstriction with diminished vasodilation in the uterine artery. However, drugs targeting the renin-angiotensin system (RAS), like losartan, are not suitable for treating preeclamptic women because they are teratogenic. Various additional experimental approaches have been used to induce preeclampsia (Marshall et al., 2018) and are not reported here. Nonetheless, it is important to emphasize that each animal model of preeclampsia can only provide insight into specific aspects of this complex disorder, underscoring the need for multiple animal models to better understand the diverse features of the disease.

Unlike non-primate animal models, non-human primates (NHPs) most closely mimic human pregnancy, exhibiting key similarities that make them highly valuable for studying mechanisms of injury and for testing vaccines and treatments aimed at preventing teratogenesis, fetal and neonatal injury, and adverse pregnancy outcomes. However, preclinical research with NHPs is challenging due to high costs, ethical considerations, and public resistance.

NHP models have been instrumental in investigating human implantation, placentation, childbirth, and endometriosis (Grigsby, 2016; Carter et al., 2015), as well as in studying the timing and effects of infectious diseases on organ development and potential alteration in neonatal behavior after birth (Coffey et al., 2018).

Angiographic studies in baboons have shed light on placental blood supply and its alterations under conditions such as experimentally induced preeclampsia *via* placental vascular restriction (Roberts et al., 2012).

The marmoset monkey, known for frequent triplet pregnancies, serves as a promising model for exploring the impact of IUGR and maternal malnutrition on placental function and fetal development (Rutherford, 2012). Additionally, the occurrence of spontaneous, multifactorial, early-life obesity in the common marmoset provides a useful model for understanding how prenatal and placental processes contribute to the developmental programming of obesity (Riesche et al., 2018).

## 4 Microphysiological systems

### 4.1 Microfluidic principles and considerations

**FIGURE 2:**
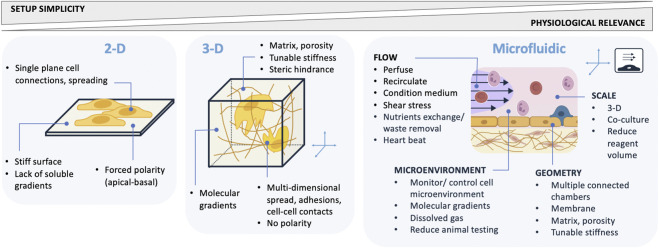
Advantages of microfluidic MPS models compared to static 2-D and 3-D models.

Microphysiological systems (MPS), including organ-on-chip (OOC) models, integrate microfluidics and cell culture to recreate dynamic, physiologically relevant microenvironments, and are specifically designed to mimic key functional features of the organ they model—such as the placental barrier for studies of maternal–fetal transport. The global benefits of dimensionality and microfluidic flow to cellular models are broad (Figure 2). Unlike conventional static 2D or 3D cultures, MPS introduce controlled flow, mechanical cues, and spatial organization that better recapitulate tissue architecture and function over cells grown in microfluidic chips. By maintaining continuous perfusion, these systems improve nutrient and gas exchange, enable the application of physiological shear stress, and allow real-time monitoring of cellular responses under tightly regulated conditions (Table 1).

**TABLE 1:** Considerations and contributions of microfluidic parameters to *in vitro* placenta models.

Parameter	Study design/feature possibilities	Technical considerations	Relevance to placental models
Flow	- Flow rate - Flow profile (pulsatile, steady) - Laminar behavior - Shear stress - Flow path recirculation - Molecular gradients	- Pump selection or gravity-driven (rocking platform, hydrostatic pressure) - Tubing and connectors, leaks and air - Basic understanding of resistance is useful	- Physical/mechanical stimuli mimic blood vessels - Flow profile to mimic physiological heartbeat - Constant and more gradual medium changes, waste removal
Microenvironment monitoring and/or control	- O_2_/CO_2_ composition - Gas gradient generation - pH stability - Temperature	- Controlled in static setups with a CO_2_ incubator - Fluidic systems may require a gas source and regulator, sensors, and consideration of gas permeability of tubing and chip	- Fetal O_2_ is lower than maternal and evolves with the stage of pregnancy
Geometry	- Mimic tissue/functional scale - Dimensionality - Compartment design - Scaling (volume, ratios)	- Commercial or home-made chips (fabrication method, resolution) - Material selection (biocompatibility, optical clarity, hydrophobicity) - Chip complexity (membrane, matrix, multiple cell types)	- Mimic a specific function or feature, e.g., FMi, placental element, molecular transport - Can scale compartment volumes, e.g., with organ volume, metabolic activity - Scale liquid volumes and membrane surface area for precise residence times
Assay automation	- Flow path control (add reagents, take samples) - Programmable steps - Versatility to select/input parameters	- Select a modular or integrated setup, commercial or home-made components - Technology and protocol standardization	- Reduce manual interventions - Increase technical reproducibility, reliability - Increase inter-operator model reproducibility, comparison of results, validation

Microfluidics lies at the core of MPS design. Flow within microscale channels is typically laminar, enabling precise control over nutrient delivery, waste removal, and gradient formation. These parameters can be tuned to mimic blood flow dynamics or interstitial transport, thereby enhancing model fidelity. Flow can be driven actively-via syringe, peristaltic, or pressure pumps- or passively, using gravity or hydrostatic pressure, depending on the required precision and complexity. Perfused systems not only sustain long-term culture but also allow monitoring and modulation of oxygen and CO_2_ levels, including the generation of hypoxic environments relevant to fetal or placental physiology.

**FIGURE 3:**
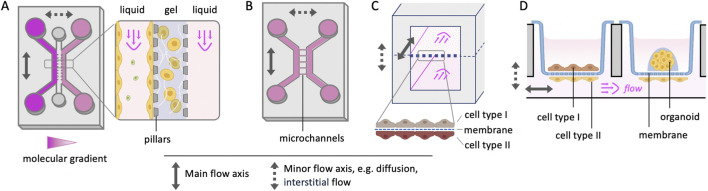
Representative architectures and options for multi-channel fluidic MPS models. **(A,B)** Multiple laterally arranged fluidic channels interconnected by a parallel gel channel **(A)** or perpendicular microchannels **(B)**. **(C)** Stacked channel design separated by a membrane. **(D)** Well plate with interconnected basal channel (modified plate base, e.g., MIROoC (Delon et al., 2025) and upper compartment (Transwell® inserts) for cell layers (*left*) or gel-embedded organoids (*right*).

The materials of MPS further influence biological performance. Polydimethylsiloxane (PDMS) remains a commonly used material due to its suitability for fast prototyping, optical transparency and gas permeability, though adsorption of hydrophobic compounds may limit pharmacological studies. Thermoplastics provide an alternative substrate with high clarity, biocompatibility and suitability for commercial-scale production, but with more limited gas permeability. Integration of sensors for real-time readouts—such as oxygen tension, glucose concentration, or barrier integrity (transepithelial electrical resistance; TEER)—enhances the analytical capability of these platforms. Increasing modularity, architecture (Figure 3), and automation are improving reproducibility and standardization, expanding MPS applicability across academic and industrial settings.

The adoption of MPS is also being accelerated by recent regulatory and ethical developments promoting the 3Rs principle (reduce, replace, refine animal use) and the implementation of New Approach Methodologies (NAMs) as alternatives to animal-based preclinical testing (Cong and Zhang, 2022; Richards et al., 2024). Very recently, recommendations from the International Council for Harmonisation of Technical Requirements for Pharmaceuticals for Human Use (ICH E21 guidelines, 2025) to include pregnant and breastfeeding individuals in clinical trials, stress the urgent need to collect appropriate data on pregnancy/breastfeeding effects, via nonclinical studies and alternative assays. Their multidisciplinary nature—combining cell biology, bioengineering, and microelectronics—positions MPS as powerful tools to investigate human-specific mechanisms that remain inaccessible *in vivo*.

In the context of reproduction, MPS provide a unique opportunity to model the human placenta—an organ that cannot be ethically or fully replicated *in vivo* or in animal models. Placenta-on-chip systems extend MPS concepts to reconstitute maternal and fetal compartments under flow, incorporating trophoblast, endothelial, and stromal components. These models aim to reproduce essential placental functions such as nutrient transport, barrier integrity, and immune modulation with unprecedented fidelity, and to offer new avenues for studying pathological conditions such as preeclampsia or intrauterine growth restriction.

### 4.2 3D placenta on-a-chip models

Placenta-on-chip models recapitulate organ-level processes such as metabolite transport and barrier function while resolving cellular-scale phenomena like differentiation and syncytialization (Table 2). These models focus on barrier integrity, trophoblast invasion and differentiation, microvilli formation, hormonal signaling, and drug/toxin transport (Elzinga et al., 2023). They are also employed to study fetal hypoxia, placental pathophysiology, pregnancy complications (e.g., preeclampsia), and preclinical therapeutic delivery, including extracellular vesicle-mediated targeting.

**TABLE 2:** Representative MPS placental models.

Model	Main application	Microfluidic setup and flow parameters	Model and contribution to placental relevance
Placenta-on-chip __ (Cao et al., 2024)	Model of transport and toxicology (nanoparticle (NP) exposure-related fetal risk)	- Setup: Transwell® plate with porous membrane culture inserts, common lower (fetal) channel - Flow: Rocking platform (10 rpm, ±8° tilt angle, bidirectional flow)	- Model: Human trophoblast stem cells (hTSCs) differentiated into STs, and human umbilical vein endothelial cells (HUVECs) on either side of a membrane. Addition of differentiated THP-1 monocytes to the hTSC side - Relevance: Model mimicked placenta villi structural architecture and barrier function. hTSCs differentiated into syncytiotrophoblasts (STB) under continuous fluid flow; Captured inflammatory response
Placenta-on-chip __ (Lermant et al., 2024)	Barrier integrity for transport assays	- Setup: OrganoPlate® (Mimetas) in CO_2_ incubator - Flow: Programmable rocking platform (7° tilt angle, 8-min cycles, bidirectional flow)	- Model: On-chip differentiation of hiPSC cells into trophoblasts - Relevance: 3-D tubule development; Structural barrier formed under flow in direct contact with an ECM gel in the absence of a physical barrier, allows environment interactions
Placenta-on-chip __ (Vidal et al., 2024)	Drugs and pollutants affecting pre-term birth (endocrine- disrupting compounds, e.g., bisphenols, and pollutants, e.g., cigarette smoke extract)	- Setup: PDMS chip made by soft lithography. Seven channels with lateral microchannel connections - Flow: Hydrostatic pressure; Gradients made using volume differentials, from 2:1 to 1.2:1, inlets: outlet; Unidirectional	- Model: Endothelial cells (PVECs, HUVECs) and primary cells (placental trophoblasts differentiated into STs, decidua, and placental stroma) in interconnected channels to create multiple interfaces (2nd trimester mimic); Addition of THP-1 macrophages in the stromal chamber - Relevance: Improved placental architecture, multicellular interactions; Captured CT invasion, endocrine production, barrier function and inflammatory response to oxidative stress
Placental barrier and FMi-on-chip __ (Safarzadeh et al., 2024)	Model pregnancy pathology and preclinical drug trial platform	- Setup: PDMS chip fabrication by soft lithography; Seven channels with lateral microchannel connections - Flow: Hydrostatic pressure. Reservoir differentials used for gradients; Unidirectional flow	- Model: Primary human fetal membrane cells (amnion epithelial, amnion mesenchymal and chorion trophoblast), decidua cells, BeWo cells to mimic placental trophoblasts (differentiated into STs), and HUVECs in interconnected channels - Relevance: Maintained intercellular interactions, interfaces, *in utero* layer thicknesses; Enabled dynamic molecular diffusion; Captured inflammatory response
Placental barrier-on-chip __ (Abostait et al., 2022)	Model of trophoblast differentiation and NP uptake (impact of flow, shear stress and trophoblast syncytialization on NP uptake)	- Setup: ibidi chip (μSlide I^0.4^ Luer) - Flow: Pressure- driven flow controller (Elveflow); Flow rate 22.9 μL/min, shear stress 0.025 dyn/cm^2^ Microvilli formation used 0.014 dyn/cm^2^; Unidirectional flow	- Model: Single channel cultured with BeWo cells; Comparison of static and in-flow conditions - Relevance: Flow promoted syncytialization and microvilli formation; Flow dynamics and degree of trophoblast syncytialization affect cell uptake of liposomes
Placental syncytium-on-chip __ (Delon et al., 2025)	Model of trophoblast differentiation (comparison of chemically versus mechanically induced syncytialization)	- Setup: Membrane- integrated recirculating organ-on-chip (MIROoC; patent pending); PET membrane between 2 stacked channels - Flow: Rocking platform (15° tilt angle, 0.3–6 rpm); Flow rate 1.5–27.5 μL/min, shear stress 0.023–0.75 dyn/cm^2^; Bi- and unidirectional flow	- Model: BeWo cell line differentiated into STs and HUVEC cultures on either side of a permeable membrane; Comparison of static and in-flow conditions - Relevance: Physiologically relevant placental syncytium-on-chip without need for chemical (forskolin)-induced differentiation; BeWo cells differentiated into STs with flow (wall shear stress 0.1 dyn/cm^2^); Captured cell fusion, polarization, barrier function, human chorionic gonadotropin secretion, and expression of key transporters
Placenta-on-chip __ (Jeong et al., 2024)	Model of early pregnancy in hypoxic environment (placenta development, trophoblast invasion)	- Setup: PDMS chip made by soft lithography; Two channels, connected by microchannels. In hypoxia chamber (2% oxygen) - Flow: Sustained perfusion not reported; Medium replaced every 12 h	- Model: Human first-trimester cytotrophoblast (HTR-8/SVneo) cell line, HUVECs lining a lumen of collagen I gel in an interconnected channel - Relevance: Improved geometry. Multi-channel 3-D model, including a vascular lumen of round cross-section; Captures tight junction formation in vessel structure, barrier function, trophoblast invasion and oxygen tension
Placenta-on-chip __ (Ghorbanpour et al., 2023)	Model of placentation in preeclampsia conditions for biomarker discovery and drug screening	- Setup: AIM Biotech chip with 3 laterally-aligned channels, made of COP thermoplastic - Flow: Interstitial flow using hydrostatic pressure gradients (volume differential of 2:1, top:bottom inlets); Medium changed every 24 h	- Model: First trimester trophoblast cell line (ACH-3P) in one side channel, HUVECs in collagen I gel in the interconnected central channel - Relevance: Representative model of the early placenta; Captures trophoblast migration and invasion and hallmarks of vascular dysfunction in preeclampsia, including upregulation of anti-angiogenesis and inflammatory- related proteins, and impaired vascular network development
Placental barrier-on-chip __ (Rabussier et al., 2023)	Model of placentation in preeclampsia and hypoxia conditions for drug transport and screening	- Setup: OrganoPlate® 3-lane 40 (Mimetas) in a low oxygen (1%) CO_2_ incubator - Flow: Programmable rocking platform (7° tilt angle, 8-min cycles, bidirectional flow)	- Model: BeWo cells differentiated into STs, and HUVECs in the outer channels, separated by central collagen I/IV layer - Relevance: Captures functional syncytium with barrier properties, polarization, secretion of relevant extracellular membrane components, thinning of the maternal-fetal space, hormone secretion, and transporter function; Captures preeclampsia features of reduced barrier function, hormonal secretion, brush border formation and increased nuclei count; Suitable for assay standardization
Implantation-on-chip __ (Park et al., 2022)	Model of FMi for trophoblast invasion and spiral artery remodeling during implantation and early pregnancy	- Setup: PDMS chip fabricated by soft lithography; Three laterally-aligned channels including a central channel for a capillary-pinned hydrogel barrier - Flow: Intentionally kept static to mimic occluded maternal vessels due to trophoblast plugs in the first trimester; Compatible with perfusion if needed	- Model: Primary extravillous trophoblasts (EVTs) isolated from first-trimester tissue, and uterine ECs in outer channels, connected *via* a collagen I channel ± decidualized primary stromal cells (DSCs) and/or uterine NK cells - Relevance: Improved 3-D microarchitecture, relative spatial arrangement of maternal and fetal elements, and maintenance of cell proliferative ability; Captures critical aspects of human implantation and early placentation such as migration of early trophoblasts towards maternal spiral arteries
Placenta-on-chip __ (Lee et al., 2016)	Model FMi for molecular transport and exchange studies	- Setup: PDMS chip made by soft lithography; Two stacked channels separated by a vitrified collagen membrane - Flow: continuous withdrawal of medium at 30 μL/h using a syringe pump	- Model: HUVECs, JEG-3 trophoblast cell line cultured on either side of a membrane - Relevance: Improved structural and functional features of FMi including co-culture, compartmentalization, dimensionality, barrier formation; Captures glucose permeability and transport
Placental organoid-on-chip __ (Wang et al., 2025b)	Model placental physiology, placenta-related gestational diseases and viral infection	- Setup: polycarbonate KabellyInsert™ chip resembling a transwell plate modified for basal channel flow and culture inserts with porous PET membranes - Flow: Rocking platform for vascular channel perfusion using gravity-driven flow (2 rpm, 6-s cycle; bidirectional)	- Model: hTSC-derived trophoblast organoids or EVT organoids made from hTSC aggregates embedded in Matrigel in the transwell insert, with HUVEC cells cultured on the membrane underside - Relevance: Improved structural and functional features of human early hemochorial placenta, including trophoblast epithelium layer and intravillous fetal capillaries, long-term trophoblast proliferation, differentiation, and viability; Captures dynamic transport in a paracrine manner, activation of innate immune-related signaling pathways and immunomodulatory factor secretion

Placenta-on-chip models typically include compartmentalized maternal and fetal chambers co-culturing trophoblasts and vascular cells, often incorporating extracellular matrix (ECM) (collagen I/IV) or semipermeable membranes. Trophoblasts may derive from hTSCs, iPSCs, primary tissue, or cell lines (BeWo, HTR-8/SVneo), differentiated into STB on- or off-chip. Endothelial cells are commonly placental villous endothelial cells (PVECs) or human umbilical vein endothelial cells (HUVECs). This architecture enables complex cellular interactions under precise biochemical and mechanical control, supporting functional studies of shear stress, tight junctions, microvilli, and transport processes (Elzinga et al., 2023; Richards et al., 2024). However, such models also have limitations as they rely on synthetic polymer membranes which biofidelity remains intrinsically low (Pemathilaka et al., 2019a).

Organoid-on-chip strategies further enhance structural and functional fidelity. Placental organoids are self-organizing 3D multicellular aggregates that more closely recapitulate maternal-fetal interactions, villi formation, and hormone secretion. Incorporating dynamic flow promotes hiPSC differentiation into cytotrophoblasts, STB, and EVT, while modulating gene expression linked to fluidic stress, Ca^2+^ signaling, and tight junction pathways (Deng et al., 2022). Co-culture with vascular cells additionally allows the modeling of inflammatory responses and antiviral defense occurring at feto-maternal interface (FMi) (Wang et al., 2025b).

While the field of placental modeling has expanded remarkably in recent years, several reviews existing reviews often prioritize specific technical or biological subsets. For example, Harrison et al. (Harrison and Bailey-Hytholt, 2025) offer a comprehensive overview of placental models, including 2D, 3D, and organ-on-chip systems, with a strong focus on trophoblast biology, chorionic villi, and models of blastocyst implantation and trophoblast invasion. Similarly, (Costa et al., 2021) present a detailed analysis of placental barrier models, particularly in the context of nutrient transport. Cherubini et al. (2021) further bridge biological and engineering perspectives by discussing both placental barrier function and trophoblast invasion. Building on these important contributions, the present review aims to complement the existing literature by positioning placenta-on-chip systems within a broader cross-scale framework. Specifically, we integrate conventional *in vitro* and *ex vivo* models with advances in microfluidic technologies and evolving regulatory considerations. We emphasize the added value of precise microenvironmental control, dynamic perfusion, and multi-compartment integration, highlighting how interconnected systems can more closely recapitulate placental physiology and support future translational and regulatory applications.

## 5 Applications of microfluidic placental models

**FIGURE 4:**
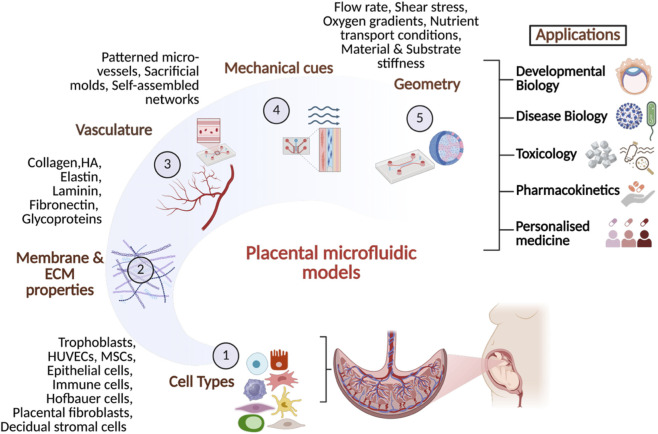
Key design considerations and applications of placental microfluidic models. This figure summarizes the principal parameters involved in engineering placental microfluidic systems, including the selection of relevant cell types, membrane characteristics, extracellular matrix composition, microchannel geometry, flow rate and shear stress, oxygen gradients, and nutrient transport conditions. Additional considerations include material biocompatibility, perfusion integration, and support for trophoblast differentiation and invasion. Potential applications span fundamental developmental biology—such as studying placental barrier function and mechanisms underlying normal and pathological placental development—as well as investigations of nutrient and drug transport, host–pathogen interactions, effects of pollutants and nanoparticles, disease modeling (e.g., preeclampsia, gestational diabetes, impaired spiral-artery remodelling), and personalized medicine approaches. Created using Biorender.com.

Microfluidic placenta-on-a-chip models offer physiologically relevant platforms to study the maternal–fetal interface. These platforms have been applied to investigate multiple aspects of placental biology. As summarized in Figure 4, these platforms enable investigations of nutrient and drug transport, infection dynamics, and pregnancy-related disorders such as preeclampsia and intrauterine growth restriction.

### 5.1 Developmental biology–the benefit of mechanistic modeling of placental development

For developmental biologists, microfluidic placental models offer experimentally tractable, human-relevant platforms that bridge cellular/molecular mechanisms and physiological transport functions. When paired with stem-cell technologies and multi-omic endpoints, these systems can reveal how defined biophysical and biochemical microenvironments drive placental development and, in turn, fetal programming making them central tools for modern studies of human developmental biology (Lermant et al., 2023). Miura et al. developed a placental microphysiological system to examine how fluid-induced shear stress influences microvilli development in human trophoblastic cells (Miura et al., 2015). Park et al. engineered a microfluidic model that simulates early-pregnancy implantation dynamics and captures the invasive behavior of EVT as they penetrate maternal uterine tissue (Park et al., 2022). Abbas et al. investigated cell migration during the implantation stage of pregnancy in a placental microphysiological system (Abbas et al., 2017). Lerment et al., demonstrated that human iPSC-derived trophoblasts, when cultured in a perfused 3D microfluidic device, self-organize into a 3D placental barrier exhibiting invasive behavior (Lermant et al., 2023). Jeong et al. studied hypoxia-induced invasion model allowing simulation of early-pregnancy placentation relevant for studying trophoblast invasion, MMP-mediated ECM degradation, spiral artery remodeling, and maternal–fetal interface formation under developmental conditions (Jeong et al., 2024). Together, these studies highlight how microfluidic placental models provide versatile, human-relevant platforms to dissect key cellular behaviors and microenvironmental cues driving placental development and early pregnancy processes.

### 5.2 Drug transfer and pharmacokinetics–the benefit of an integrated selective membrane

Microfluidic placenta-on-chip devices enable precise analysis of maternal-fetal drug transfer under dynamic flow conditions, providing greater physiological relevance than traditional static systems. By reproducing essential microenvironmental features—such as shear stress, cellular architecture, and selective barrier properties—these models provide more accurate assessments of drug transport and barrier function. A representative study demonstrated glucose transport using co-cultured BeWo b30 trophoblast cells and human placental villous endothelial cells (HPVECs) on a fibronectin-coated polycarbonate membrane within a PDMS platform, achieving a glucose transfer rate of 34.8%, closely approximating values observed in *ex vivo* perfused placentas and outperforming static transwell models (Blundell et al., 2016). Another study investigated glucose transport under malarial infection using a placenta-on-a-chip model. Co-culturing BeWo and HUVECs on collagen I–coated membranes revealed that chondroitin sulfate A–adherent malaria-infected erythrocytes increased barrier resistance and reduced glucose transport compared with uninfected controls (Mosavati et al., 2022; Mosavati et al., 2020).

The transfer of naltrexone and its metabolite 6β-naltrexol, used in opioid addiction therapy, has been examined using a similar setup with BeWo cells and HUVECs cultured on an entactin–collagen IV–laminin–coated polyethylene terephthalate (PET) membrane within a PDMS platform. Only ∼2.5% and ∼2.2% of maternal concentrations reached the fetal channel, significantly lower than the ∼10% transfer observed in acellular controls (Pemathilaka et al., 2022). A comparable model assessing heparin and glyburide transport showed ∼95% glyburide maternal retention due to active BCRP-mediated efflux and minimal fetal transfer (∼5–6%), consistent with *ex vivo* human placenta data (0.6%–3.9%). No heparin was detected on the fetal side after maternal administration, underscoring the selective regulatory role of trophoblasts (Blundell et al., 2018). Similarly, the kinetics of statin transport (rosuvastatin and pravastatin) evaluated in a co-culture of BeWo and HUVEC on collagen I–coated membranes within PDMS systems demonstrated both compounds crossing the placental barrier within 8 h (Richardson et al., 2022). Ahn et al. investigated the effect of levonorgestrel on a microfluidic device that can replicate the endometrial microenvironment (Ahn et al., 2021). Collectively, these studies illustrate the ability of placenta-on-a-chip models to capture dynamic pharmacokinetic behavior and drug-specific transfer mechanisms, offering a physiologically relevant alternative to conventional assays.

Among the limitations of known solutions one can observe that static *in vitro* cell cultures such as transwell-like setups (e.g., parallel artificial membrane permeability assay (PAMPA) with human colon adenocarcinoma Caco-2 cells) still cannot mimic the multi-layered structure and functionality of the placental barriers, even with polarized environments (Bailey-Hytholt et al., 2020). In this aspect, they are failing to recapitulate endothelial contribution and placental dynamics to physiological barrier function. Such systems by their nature enhance their nascent signal/noise ratio with different surface areas, cell quantities, and static media volumes between double chambers, leading to artificial negative results in these experiments. Indeed, PAMPA retains elements of a more traditional static assay, because even though it uses a membrane, molecules move across by passive means such as diffusion. Missing from these transwell-like assays are controlled vascular-like flows and other active mechanical forces that microfluidic setups provide to improve the physiological relevance of models. Readouts such as drug permeability are also reported using different equations, too few time points and variable test conditions, which together with assumptions of the first-order reaction and passive diffusion only, make it difficult to compare with other studies and clinical data.

The choice of materials used in construction of these systems (Blundell et al., 2016; Pemathilaka et al., 2019b) commonly relies on polydimethylsiloxane (PDMS), polytetrafluoroethylene (PTFE) or polycarbonate plastic membranes as surrogates to placental tissues properties (seeding cells on both sides on the membrane and placing it into a MPS). This does not fulfil the required matching of combined physical, chemical and biomechanical properties and functionality of the placental barrier (e.g., PDMS has shown to reduce the pharmacological and drug absorption reactions, because of consuming hydrophobic molecules).

There are also other challenges in assessment of the drug transport in placental MPS. Analysis of the drug transport foresees sufficiently detailed understanding of mechanisms, stages of molecular interactions, and transporters. The complexity of associated differential equations requires too many assumptions leading at the end to over-simplifications. Some reported placental MPS require weeks of culture preparation and more for the experiment duration, leading to a large data scatter and difficult-to-use endpoints (e.g., it is known that antipyrine clearance depends on blood flow dynamics but such factors in the published tests results are either fixed or not clearly reported). Automated microfluidic setups enable the collection of time-resolved data points for the capture of dynamics. Observed large lag times (>10–30 min) and reported test drug concentrations 2-3-fold higher than those originally administered (in contrast to what mass conservation law allows), impose that a caution should be taken to process such data for validation (Jacobsen et al., 2023; Masserdotti et al., 2024).

### 5.3 Toxicology and Nanotoxicology–the benefit of flow

Microfluidic placental barrier models based on dual perfused channels separated by a 3D extracellular matrix that mimic the multilayered maternal-fetal interface, enable high-fidelity assessment of nanoparticle and microparticle toxicity by capturing flow-dependent effects on barrier integrity and transport function. Exposure to titanium dioxide (TiO_2_) nanoparticles in a BeWo–HUVEC co-culture on a matrigel–chitosan-coated membrane induced oxidative stress, apoptosis, and barrier dysfunction, and notably triggered the attraction of maternal macrophages by trophoblast cells, a response associated with further impairment of placental barrier function (Yin et al., 2019). Similarly, a microfluidic barrier model employing a collagen I–coated semipermeable polyethylene terephthalate membrane positioned between maternal and fetal PDMS channels demonstrated dose-dependent trophoblast cell death upon exposure to carboxyl-modified polystyrene microparticles, although overall barrier integrity was preserved (Boos et al., 2021). Gresing et al. used a commercial microfluidic device model to study the transport of magnetic NPs under continuous flow conditions in a time-dependent manner (Gresing et al., 2021). Abostait et al. investigated the impact of nanoparticle exposure on trophoblast syncytialization and microvilli formation under dynamic conditions (Abostait et al., 2022). Advanced 3D PDMS-based platforms integrating extracellular matrix components, intraluminal flow, and fluorescently tagged trophoblasts now allow real-time imaging and quantification of invasion dynamics (Pu et al., 2021; Shojaei et al., 2021). Moreover, combined shear stress and syncytial differentiation have been shown to enhance chondroitin sulfate A–liposome uptake, emphasizing the need for dynamic physiological conditions in nanoparticle transport studies (Abostait et al., 2022). Together, these findings underscore the capacity of microfluidic systems to model dose-response relationships, nanotoxic effects, and immunological interactions within a controlled and reproducible environment.

In general, a more complete and detailed reporting of experimental microfluidic setup and parameters will be important for wider comparison, validation and acceptance of fluidic models. Many placenta-on-chip studies do not report using continuous flow and/or provide insufficient details to enable precise replication of experimental conditions in another lab. Generating hydrostatic pressure differences by unevenly filling reservoirs or using standard rocking platforms is an accessible entry point to dynamic experiments, but makes flow conditions somewhat difficult to define, control and reproduce. Indeed, the effects of flow rate, pulsatility profile, flow directionality (uni- or bidirectional) and shear stress on cellular models are not yet well understood. Some studies are starting to address flow directionality and shear stress and reporting direct comparisons with static controls (see Table 2), which are important in validating these new models and understanding the relevance and ranges of specific mechanical and fluidic parameters.

### 5.4 Infection modeling–the benefit of co-cultures and connected systems

Placenta-on-chip systems provide a promising platform to study maternal–fetal pathogen transmission and associated immune responses. A 3D microfluidic placenta–fetus model incorporating HTR8/SVneo trophoblasts, HUVECs, and fetal neural progenitor cells demonstrated that Zika virus can traverse the placental barrier and infect downstream neural tissue, causing fetal cell death—an effect mitigated by chloroquine treatment. The presence of placental cell layers significantly reduced viral transmission compared to acellular controls (Arumugasaamy et al., 2018). In another study, *E. coli* exposure in BeWo–HUVEC co-cultures on collagen I–coated membranes triggered inflammatory cytokine secretion and enhanced macrophage adhesion (Zhu et al., 2018). Another group studied ascending infection of *E. coli* from maternal to fetal tissue using primary cells from the decidua, chorion, amnion mesenchyme and amnion epithelium and collagen rich matrix from full-term patients into a four-chamber co-culture model (Richardson et al., 2020). More recently, microfluidic models employing iPSC-derived trophoblasts and endothelial cells revealed that STBs are highly susceptible to SARS CoV-2 infection, with ∼57% infection at day 3, leading to impaired STB differentiation, reduced fusion index, and diminished HCG secretion (Chen et al., 2023). These examples highlight the utility of organ-on-chip systems in dissecting infection mechanisms, barrier responses, and antiviral interventions in a physiologically relevant context.

### 5.5 Disease modeling and personalized screening–the benefit of microenvironment control

Microfluidic placental models have been applied to study pathologies linked to adverse fetal development including preeclampsia, gestational diabetes, placental malaria, impaired spiral-artery remodeling, fetal growth restriction, stillbirth, and to test candidate therapies or targeted delivery systems with fetal-safety readouts. Preeclampsia is a complex pregnancy disorder affecting 2%–8% of pregnancies and is characterized primarily by hypertension, proteinuria, hypoxia/reoxygenation, oxidative stress, inflammation, and disruptions in maternal and fetal immune system functions (Jena et al., 2020). Understanding its pathogenesis is crucial for developing effective diagnostic and therapeutic strategies (Knyazev et al., 2025). Models co-culturing ACH-3P trophoblasts and HUVECs on collagen I–coated membranes have demonstrated that TNF-α-mediated inflammation upregulates FKBPL and Gal-3, disrupts vascular network formation, and replicates key hallmarks of preeclampsia (Ghorbanpour et al., 2023). Functional placenta-on-a-chip models using BeWo b30 and HUVECs cultured on collagen I/IV matrices reproduced essential physiological characteristics, including syncytium formation, barrier integrity, hormone secretion, and active transporter function (Rabussier et al., 2023). This model exposed to low oxygen conditions and perfusion flow shows the pathological characteristics of preeclamptic placentas (reduced barrier function, hormone secretion, and microvilli count) (Rabussier et al., 2023). Several groups have also explored the effects of hypoxic (0.5% O_2_) vs. normoxic (21% O_2_) conditions on trophoblast migration to elucidate oxygen-driven mechanisms underlying preeclampsia pathogenesis (Cho et al., 2021; Ko et al., 2022; Elzinga et al., 2023; Jeong et al., 2024).

Utilizing hiPSCs enables the recreation of patient-specific processes. hiPSCs can be easily derived from individuals, reducing ethical concerns compared to human embryonic stem cells (hESC). For example, patient-derived models, such as hiPSC-derived trophoblast models have proven valuable in studying pregnancy complications like preeclampsia (Sheridan et al., 2019). Recent research shows that hiPSC-derived TSC and their EVT derivatives retain epigenetic memory of the pre-eclamptic placenta, exhibiting gene expression changes and hypermethylation patterns linked to trophoblast invasion and ECM organization (Morey et al., 2024), further emphasizing their potential for personalized therapeutic approaches. By combining these patient-derived cellular sources with advanced microphysiological platforms, it becomes possible to model disease mechanisms in a personalized manner, offering a level of individual specificity that goes beyond conventional culture systems and significantly enhancing the translational potential of preeclampsia research.

## 6 Conclusion

Microfluidic models have significantly advanced placental research, helping to bridge the gap between traditional *in vitro* approaches and *in vivo* studies. By combining dynamic flow, co-cultures of different cell types, and controlled microenvironments, placenta-on-a-chip platforms make it possible to study nutrient exchange, barrier properties, immune interactions, and disease mechanisms in conditions that are closer to human physiology. Compared to conventional systems, these models allow better control and reproducibility, as well as real-time monitoring, while reducing the need for animal experimentation.

Despite this progress, several challenges remain. Current models still lack standardization and often cannot fully reproduce the cellular and mechanical complexity of the placenta. Differences in materials, chip formats, flow control systems, and experimental setups can also affect reproducibility and physiological relevance, making difficult to compare results between studies. Systematic evaluation of parameters such as flow rate, shear stress, and organ connectivity is essential, particularly for multi-organ platforms. Efforts by initiatives such as the European Organ on Chip Society (EUROoCS) and International Microphysiological Systems Society (IMPSS) aim to define technical standards and workflow protocols, while benchmarking against clinical and preclinical data will be key for translational adoption.

Beyond technological refinement, an important next step is the integration of placental MPS into regulatory science. The development of robust, fit-for-purpose placenta-on-chip platforms that accurately replicate key aspects of placental functionality has the potential to contribute to global regulatory strategies that address the use of drugs in pregnancy. Recent guidance, including the International Council for Harmonisation (ICH) of Technical Requirements for Pharmaceuticals for Human Use E21 (2025) *Inclusion of Pregnant and Breastfeeding Individuals in Clinical Trials*, emphasizes the need for proactive planning and early data generation to inform the safe use of investigational products during pregnancy and breastfeeding. However, real-world human data particularly relating to first-trimester placental function and early maternal–fetal health remain extremely limited. In this context, well-validated placental MPS could represent a transformative tool for generating mechanistic and exposure-relevant data to complement clinical and epidemiological evidence.

Such systems may also support post-marketing safety monitoring. As highlighted in ICH E21, rare or delayed adverse pregnancy outcomes are unlikely to be fully captured in pre-authorization clinical trials. Human-relevant placental MPS could therefore be incorporated into investigational and post-approval frameworks to help interpret safety signals, explore mechanisms of toxicity, and prioritize further clinical or pharmacovigilance investigations.

From a nonclinical safety perspective, alignment with evolving reproductive and developmental toxicity (DART) testing strategies will be critical. The ICH S5 (R3) guideline, Detection of Reproductive and Developmental Toxicity for Human Pharmaceuticals, encourages the use of alternative and mechanistically informative assays, including advanced *in vitro* systems, particularly when assessing the effects of human-relevant metabolites. This is especially important given the well-recognized differences in DART outcomes between animal models and humans, as noted in ICH M3 Annex 1. Standardized placental MPS platforms, once qualified, could therefore complement existing DART packages by providing human-specific insights into placental transfer, metabolism, and toxicity.

Looking ahead, progress toward regulatory acceptance will require coordinated efforts in standardization, inter-laboratory reproducibility studies, definition of context-of-use, and formal qualification within regulatory frameworks. In parallel, future technological developments will likely focus on more integrated and personalized systems. For example, linking placenta chips with other organ models—such as uterine, fetal, or hepatic tissues—could improve understanding of systemic maternal–fetal interactions and drug disposition during pregnancy. The convergence of stem cell technologies, bioengineering, and regulatory science will be essential to transition these platforms from experimental tools to qualified models with real predictive value for maternal and fetal health.
